# Bladder Tumor in Women with Microscopic Hematuria: An Iranian Experience and a Review of the Literature

**DOI:** 10.1155/2009/231861

**Published:** 2009-07-20

**Authors:** Shahin Abbaszadeh, Saeed Taheri, Mohammad Hossein Nourbala

**Affiliations:** ^1^Dr. Taheri Medical Research Group, Baqiyatallah Research Center for Gastroenterology and Liver diseases, Baqiyatallah Hospital, Mullasadra Ave, Tehran 1435915371, Iran; ^2^Department of Urology, Baqiyatallah University of Medical Sciences, Tehran 1435915371, Iran

## Abstract

*Aim*. In this study we report our experience with microhematuria and its relation with bladder tumors in Iranian women. *Materials and Methods*. Overall 249 women were evaluated. Microscopic hematuria was defined as three or more red blood cells per high-power field on at least two different occasions. Patients with a history of gross hematuria or coagulation disorders, having organic diseases, urinary stones, urinary tract infections, nephrological diseases, and local lesions such as urethral caruncle were excluded from the study population. Final diagnosis of malignant tumors was done with cystoscopy and biopsy specimen pathological assessment in all cases. *Results*. Age for the study population was 49.7 ± 11.8 years. 95 (38%) of patients were identified during routine check up and presenting symptoms in other patients were frequency, dysuria, stress urge incontinence, urge incontinence, feeling of incomplete urine emptying, and flunk pain, respectively. Finally, 7 (2.8%) of study subjects were confirmed as having bladder tumors. One of tumor cases was diagnosed 24 months after initial assessments. Patients with bladder tumor were significantly older; more frequently had diverticulum in their bladder wall (*P* < .05). *Conclusion*. Female microscopic hematuria is relevant and deserves evaluations, especially in elderly patients. Patients whose reason for microhematuria would not be diagnosed at the initial evaluations should be followed.

## 1. Introduction

Microscopic hematuria is a common clinical problem with a prevalence of 0.18 to 21 percentin adults [1–5]. This wide range reflects the differing criteria for defining clinically significant haematuria, and the varying ages of the study population. Urine normally containsa few red blood cells and microscopic hematuria is generally defined as three or more red blood cells per high power field (HPF), from at least two properly collected urinalysis specimens [6, 7]. 

 Microscopic hematuria may be transient. It may occur in up to 13% of postmenopausal women [8]. Transient microscopic hematuria in women may be caused by sexual intercourse, by menstrual contamination, or even by mild trauma. Microscopic hematuria, unlike gross hematuria, is often an incidental finding, but may be associated with serious conditions. The many causes range from insignificant lesions (caused by, e.g., vigorous exercise) to highly significant life-threatening lesions (e.g., urologic malignancy in up to 10% of adults) [9]. 

 Microscopic hematuria in women may be of lesser relevance than in men [6] but, there is still need for further evaluations in women with microscopic hematuria [10–12]. Different incidences of bladder tumors among women with microhematuria have been reported from different countries. In this study, we aimed to assess the incidence of bladder tumor among Iranian women undergoing investigation for microscopic hematuria.

## 2. Methods and Patients

### 2.1. Patients

Microscopic hematuria was defined as three or more red blood cells per high-power field on at least two different occasions, with a minimum time gap of at least two weeks between. We included all 249 female patients, without a previous history for urologic problems, who were detected incidentally at our outpatient clinic or were referred to our clinic for the evaluation of microscopic hematuria, from February 2003 through March 2007. All referred patients were reevaluated for confirmation of having hematuria. 

 Urine cytology was performed in a number of patients (depending on physician's decision with no definite criteria) to evaluate whether a neoplastic process was the source of the hematuria before any radiological or urologic procedures; mid-stream urine samples were collected, immediately processed, and subsequently examined cytologically. Urinalysis and urine culture were performed on all patients. Patients with a history of gross hematuria or coagulation disorders, having organic diseases, urinary stones, urinary tract infections, nephrological diseases, and local lesions such as urethral caruncle were excluded from the study population. 

 Renal ultrasonography and cystoscopy were performed on all patients. If the patients had persistent hematuria with no definitive diagnosis, Intravenous urography (IVU) was performed. 

 Before cystoscopy, vaginal and urethral examinations were performed in all patients to exclude local causes of microscopic hematuria. Rigid cystoscopy was done under local anesthesia. At cystoscopy urethra, urethral orifice and systematic evaluation of the entire bladder surface was performed. Cystoscopy was done by using the 30 and 70 degree lens. After complete inspection of the urethra and bladder has been accomplished, the bladder is drained and the instrument is gently removed. Biopsy was performed if suspicious lesions were seen or if (+) cytology was obtained. Final diagnosis of malignant tumors was done with cystoscopy and biopsy specimen pathological assessment in all cases.

### 2.2. Statistical Analysis

Data are shown as mean ± SD. Pearson Chi-square test and student's *t*-test were used for evaluations where appropriate. Non-parametric evaluations by Chi-square and Mann-Whitney *U*-tests were also performed for confirmation; non parametric evaluations did not change initial findings. Because of the limited number of patients included into this study, we were unable to make multivariate analyses. Two-tailed *P* values ≤ .05 were considered significant.

## 3. Results

Age for the study population was 49.7 ± 11.8 years (range 30–85). 95 (38%) of patients were identified during routine check up; presenting symptoms in the remaining 154 (61.8%) patients were frequency 61 (24.5%), dysuria 51 (20.5%), stress urge incontinence 39 (15.7%), urge incontinence 24 (9.6%), feeling of incomplete urine emptying 23 (9.2%), and flunk pain 7 (2.8%). On ultrasonography, 12 (4.8%) had large urinary residual volumes. 

 Cystoscopic evaluations revealed 10 patients with urethral stricture, 83 (33%) with mild to moderate and 3 (1.2%) with severe bladder wall trabeculation. Three (1.2%) also had bladder wall diverticulum. Overall, 7 (2.8%) patients were diagnosed as having bladder tumor. The characteristics of these patients are summarized in Table 1. Patients with bladder tumor were significantly older than others (67.6 ± 12.2 versus 49.1 ± 11.4 years old, resp. *P* < .0001). There was no difference between two groups (with and without malignancy) regarding their presenting symptoms. Patients with bladder tumor had significantly higher rate of diverticulum (*P* = .001).

## 4. Discussion

In our study, we found a 2.8% bladder tumors frequency among our Iranian females evaluated for microhematuria. Other similar studies have shown both higher and lower rates. No cases of bladder cancer were found in one prospective, referral-based study involving 177 American women (mean age, 57.2 years) with asymptomatic microscopic hematuria who underwent cystoscopy [13]. In a similar study, involving 1034 Japanese adults (75% female), only two cases of bladder cancer was identified in women with microscopic hematuria [14]. Another study conducted in the UK revealed an incidence of 1.4% of bladder tumors in patients with microhematuria [15]. On the other hand, some other studies have also reported high incidences of malignancy among patients with microscopic hematuria. In a study on 100 Bengali patients undergoing evaluations for microscopic hematuria, an astonishing incidence of 16 (16%) malignancy (bladder tumor in 10 (10%)) was observed [16]. In a report from the UK, 51 out of 982 patients (5.2%) presenting with microhematuria were diagnosed as having cancer [17]. A Swedish study on patients with hematuria demonstrated an incidence of 9% malignancies; 5% when asymptomatic [18]. 

 The reasons behind these disparities may vary substantially. Higher incidence of bladder cancer in specific ethnic groups may be related to different causes from genetic factors to particular lifestyle specificities such as food habits and cigarette use. On the other hand, some interventional factors may also confuse study results. Different characteristics of the study subjects in terms of age and gender and use of different inclusion criteria and different study designs (e.g., prospective, longitudinal, and retrospective and/or population based versus referral based studies) can play significant roles in the study end results. There have been only a few population-based studies addressing the incidence of microscopic hematuria. Some studies suggest that there may be an increased incidence among older persons, some others show no difference according to age [8] and two studies reported a higher incidence among women than among men [10]. 

 In our study, the incidence of malignancy was strongly associated with patients' age (*P* < .0001). Previous studies also strongly corroborate this finding. No female under 70 years and no male under 45 years of age with microscopic hematuria was found to have malignant bladder tumors, in a study by Boman et al. in Sweden [18]. An American study assessing incidence of malignancy among individuals with microhematuria also reported that none of women with cancer were below 65 years (versus 3/11 men) [19]. However, in our study, 2 out of 7 cases of bladder tumor were below 65 years of age and the youngest case was 49 years old (Table 1). 

 Gender plays a significant role in the incidence of bladder tumors. Most previous studies have demonstrated a higher incidence of bladder tumor among males with a reported male to female ratio of 2.7 : 1 [18–20]. Jaffe et al. reported that 3 women out of 62 (4.8% versus 11 (11.2%) men out of 98) with microhematuria who underwent cystoscopy and ultrasonography represented bladder cancer [19]. Boman et al. also found that the incidence of malignancies was strongly sex-related and occurs predominately in males [18]. According to these findings, some authors declared that microscopic hematuria in females is of lesser relevance than in men [6], especially when the women are under 65; however, our study, with a 2.8% frequency of bladder tumor detection in women with microhematuria, of whom about 29% were below 65 years of age, suggests that women with microscopic hematuria, even under 65 years of age, should also receive full evaluations. 

 The initial determination of microscopic hematuria should be based on microscopic examination of urinary sediment from a freshly voided, clean-catch mid stream urine specimen [21]. The recommended definition of microscopic hematuria is three or more red blood cells per (HPF) on microscopic evaluation of urinary sediment from two of three properly collected urinalysis specimen [17, 21]; however, some investigators had a more rigid definition of equal to or greater than 1 red blood cell per HPF as significant hematuria [9, 22–24]. Some others have defined a threshold of 6 or more RBCs per HPF as significant hematuria [25, 26]. 

**Figure 1:**
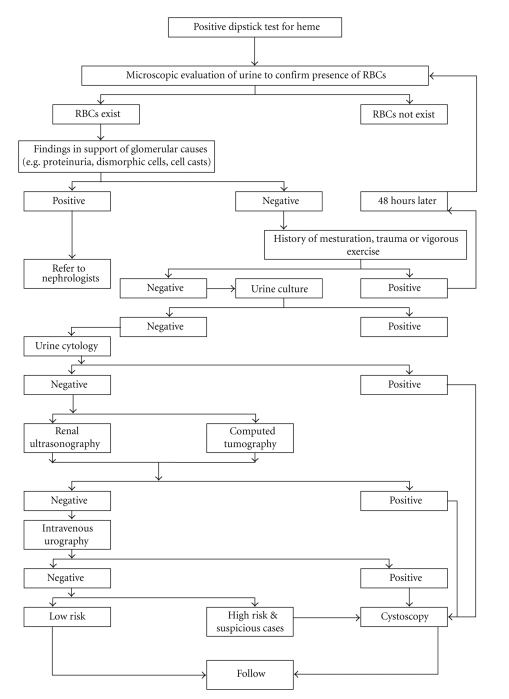
Algorithmic approach to microscopic hematuria in females.

 An algorithmic approach to the workup of microhematuria in females is shown in Figure 1. The presence of significant proteinuria, red cell casts, or predominance of dysmorphic red blood cell in the urine should prompt an evaluation for renal parenchymal disease or referral to a nephrologist. 

 If a careful history suggests a potential benign cause for microscopic hematuria, the patients should undergo repeat urinalysis 48 hours after cessation of the activity (menstruation, vigorous exercise, sexual activity, or trauma). No additional evaluation is warranted if the hematuria has resolved. Patients with persistent hematuria require evaluation. In women, urethral and vaginal examination should be performed to exclude local causes of microscopic hematuria. Patients with urinary tract infection should be treated appropriately and urinalysis should be repeated six weeks after treatment. If the hematuria resolves with treatment, no additional evaluation is necessary. Cystoscopy as a component of initial evaluation of microscopic hematuria in all adult patients more than 40 years of age, and in patients less than 40 years of age with risk factors for bladder cancer, (e.g., current or former tobacco users; persons with occupational exposure in chemical, textile, or rubber industries; persons with past exposure to phenacetin, cyclophosphamide, or pelvic radiation therapy; patients with chronic urinary tract infections or neurogenic bladder; patients with spinal cord injury with intermittent catheterization or indwelling catheter) [27]. 

 One of our cases was diagnosed as having bladder tumor almost 24 months after initial evaluations for microhematuria (Case 3). During this time period, she underwent repeated cystoscopy and cytological evaluations and pathological studies on biopsy specimens; however, nothing related to malignancy was found. Chow et al. also reported that 1% of patients would represent transitional carcinoma of urinary bladder 20 months after initial presentation [28]. Messing et al. found a less than 9 months brief preclinical duration of bladder tumors for men [29]. 

 In conclusion, microscopic hematuria in females, either symptomatic or asymptomatic, is of extreme relevance and deserves full evaluations; especially in elderly patients or patients at high risk for bladder cancer. Here we highlight the finding that only 1 of the 249 patients had true symptomatic microhematuria and was found to have cancer. Patients whose reason for microhematuria would not be diagnosed at the initial evaluations should be followed.

## Figures and Tables

**Table 1:** Characteristics of patients with confirmed diagnosis of bladder tumor.

Patients	Presenting symptoms	Age	Ultrasonography	Tumor-stage	Grade	Other findings
Case 1	Check up	49	Single tumor on the lateral bladder wall	TCC-T1	Grade I	Cystoscopy: tumor on the right lateral bladder wall, superior to ureteral orifice; size: 1∗2 cm
Case 2	Right flunk pain	70	Thickening of the bladder wall, single tumor	TCC-Ta	Grade III	IVU: diverticulum inside the right lateral bladder wall; size: 3∗2 cm
						Cystoscopy: tumor inside the diverticulum, superior to right ureteral orifice
Case 3	Suprapubic pain and frequency	78	3 ∗ papillary tumors	TCC (diagnosed 24 months after initial evaluations)-Ta	Grade II	Cystoscopy: mild bladder trabeculation and inflammation and 3 Tumors, two on left bladder wall superior to the ureteral orifice and one on the floor of bladder; sizes: 1∗1 & 1∗0.5 & 1∗2 cm
						Cytology: negative for tumor (3 times during 24 months)
Case 4	Disuria	60	Single papillary tumor	TCC-T1	grade II	Cystoscopy: tumor on the right lateral bladder wall, superior to ureteral orifice; size: 2∗3 cm
Case 5	Disuria, urinary frequency, urge incontinence, stress urge incontinence	70	Single tumor	TCC-T1	grade II	Cystoscopy: tumor on the left bladder wall; size: 2∗2 cm
Case 6	Disuria and frequency	68	Single tumor	TCC-T1	Grade I	Cystoscopy: tumor on anterior bladder wall; size: 2∗2 cm
Case 7	Disuria, frequency, urge incontinence	85	Single tumor	TCC-Ta	grade I	Cystoscopy: Tumor on the right lateral bladder wall; size: 2∗2 cm
